# Neck pain patterns and subgrouping based on weekly SMS-derived trajectories

**DOI:** 10.1186/s12891-020-03660-0

**Published:** 2020-10-14

**Authors:** P. Irgens, A. Kongsted, B. L. Myhrvold, K. Waagan, K. B. Engebretsen, B. Natvig, N. K. Vøllestad, H. S. Robinson

**Affiliations:** 1 Department of Interdisciplinary Health Sciences, Institute of Health and Society, University of Oslo, P.O. Box 1089, Blindern, 0317 Oslo, Norway; 2 Department of Sports Science and Clinical Biomechanics, University of Southern Denmark, Odense, Denmark; 3 Nordic Institute of Chiropractic and Clinical Biomechanics, Odense, Denmark; 4 Department for Data Capture and Collections Management, University Center for Information Technology, University of Oslo, Oslo, Norway; 5 Department of Physical Medicine and Rehabilitation, Oslo University Hospital, Oslo, Norway; 6 Department of General Practice, Institute of Health and Society, University of Oslo, Oslo, Norway

**Keywords:** Neck pain, Clinical course, Subgroup, Longitudinal, Episodes, Fluctuations, Chiropractic, Back pain

## Abstract

**Background:**

Neck and low back pain represent dynamic conditions that change over time, often with an initial improvement after the onset of a new episode, followed by flare-ups or variations in intensity. Pain trajectories were previously defined based on longitudinal studies of temporal patterns and pain intensity of individuals with low back pain. In this study, we aimed to 1) investigate if the defined patterns and subgroups for low back pain were applicable to neck pain patients in chiropractic practice, 2) explore the robustness of the defined patterns, and 3) investigate if patients within the various patterns differ concerning characteristics and clinical findings.

**Methods:**

Prospective cohort study including 1208 neck pain patients from chiropractic practice. Patients responded to weekly SMS-questions about pain intensity and frequency over 43 weeks. We categorized individual responses into four main patterns based on number of days with pain and variations in pain intensity, and subdivided each into four subgroups based on pain intensity, resulting in 16 trajectory subgroups. We compared baseline characteristics and clinical findings between patterns and between Persistent fluctuating and Episodic subgroups.

**Results:**

All but two patients could be classified into one of the 16 subgroups, with 94% in the Persistent fluctuating or Episodic patterns. In the largest subgroup, “Mild Persistent fluctuating” (25%), mean (SD) pain intensity was 3.4 (0.6) and mean days with pain 130. Patients grouped as “Moderate Episodic” (24%) reported a mean pain intensity of 2.7 (0.6) and 39 days with pain. Eight of the 16 subgroups each contained less than 1% of the cohort. Patients in the Persistent fluctuating pattern scored higher than the other patterns in terms of reduced function and psychosocial factors.

**Conclusions:**

The same subgroups seem to fit neck and low back pain patients, with pain that typically persists and varies in intensity or is episodic. Patients in a Persistent fluctuating pattern are more bothered by their pain than those in other patterns. The low back pain definitions can be used on patients with neck pain, but with the majority of patients classified into 8 subgroups, there seems to be a redundancy in the original model.

## Background

Neck pain (NP) and low back pain (LBP) are costly, common, and among the health conditions with the highest impact on disability across the world [1]. Evidence on the clinical course of spinal pain challenges the common understanding of spinal pain defined as acute, sub-acute or chronic conditions [2], and being categorized as recovered or non-recovered [3, 4]. Instead, spinal pain seems to represent dynamic conditions that change over time. In reality, the clinical course is mostly characterized by an initial improvement after the onset of a new episode, followed by flare-ups or more persistent patterns of variations in intensity or episodes [5–8].

In a review paper, a collaborative group of LBP researchers concluded that trajectory studies on LBP are numerous and have identified similar trajectory patterns [2]. From a theoretical standpoint, it is difficult to see that future studies will uncover considerable changes in existing trajectories. However, facilitating common terminology and categorization criteria for the patterns and subgroups will help promote consistency in the field of subgroup research. Also, there is a need for assessments on the number of classes that are clinically useful and recognizable. The collaborative group advised that focus should be on subgroups constructed on a combination of pain variation patterns, pain intensity, and speed of improvement based on previously identified trajectories [2]. To further investigate the usefulness of these variation patterns, it was also recommended to test whether the findings on LBP are similar across cohorts and conditions.

Kongsted and coworkers defined 16 subgroups based on two of the suggested constructs: pain variability and pain intensity, by outlining 4 standardized definitions of variation patterns (Ongoing, Fluctuating, Episodic and Single episode) [9]. These 4 patterns were further separated into 16 subgroups based on pain intensity levels (Severe, Moderate, Mild and Minor), and subsequently applied to a Danish cohort with LBP [9]. Classifications of patients using these definitions matched well with latent class analysis-derived trajectory patterns from the same cohort.

The definitions have so far only been applied to LBP patients. There are only two clinical course studies on NP for comparison [6, 10]. This limits the possibility of producing similar collaborative definitions as for LBP. However, previous studies show similarities between the clinical course of NP and LBP [5, 6, 11]. In addition, patients with NP and LBP have several similarities in psychosocial prognostic factors and comorbidities, clinical guidelines for best practice, and lack of specific pathoanatomic causality [11–14]. While the models for clinical management of musculoskeletal complaints to date have mainly been condition specific, there have recently been calls for management based on characteristics within the biopsychosocial model regardless of pain condition [14–17]. Studies have also demonstrated that patients with trajectories of NP display similar on most health-related factors as for LBP [5, 6]. Thus, as a next step in subgroup development it is important to examine how well the definitions based on LBP will fit in a NP cohort, and if the group of patients in the patterns differ with regards to clinical characteristics.

The objectives of this study were to 1) investigate if the defined patterns and subgroups for LBP are applicable to NP patients in chiropractic practice, 2) explore the robustness of the defined subgroups, and 3) investigate if the patients in the defined patterns or subgroups differ with respect to baseline characteristics and clinical findings.

## Methods

### Study design and setting

This study was part of a prospective, observational study on patients with NP in chiropractic care setting. Members of the Norwegian Chiropractic Association were invited to participate in the recruitment of patients. We asked seventy-two chiropractors geographically spread in Norway to invite all consecutive patients with NP from September 2015 until June 2016 to participate in the study. The chiropractors gave interested patients written and verbal information about the study. Patients that accepted to participate signed a written consent. The study was approved by The Norwegian Regional Committees for Medical and Health Research Ethics (2015/89).

We invited patients aged 18 years or more presenting with, or already in a treatment course for, a bothersome neck as a primary or secondary complaint with or without radiating arm pain to participate. They were eligible for inclusion regardless of pain duration and time since last chiropractic treatment. Patients had to possess and be able to operate a mobile phone and have basic Norwegian reading and writing skills. They were not included if serious pathology was suspected (inflammatory or pathological cause, fracture, or radiating pain requiring acute surgery). All patients received standard chiropractic care at the discretion of the chiropractor, unaffected by inclusion in the study.

### Data collection

Patients completed a self-administered questionnaire at baseline, 4, 12, and at 52 weeks, either on paper or digitally. The present study used questionnaire data from baseline. Additional descriptions of recruitment of the cohort, the procedures and the questionnaires have been published previously [18, 19]. A researcher (PI or BLM) or an assistant contacted the patients by telephone to provide further information regarding the study procedures. Once a week, at the same day and time over a 52-week period, the patients received 2–3 automated short message services (SMS) with the following questions (Additional file 1): “How many days the last week has your neck been bothersome? Please answer with a number between 0 and 7” (hereafter ‘paindays’). If the answer to the first SMS was 0, question 2 was not sent. If the answer was between 1 and 7, the patient received a second SMS “How intense has your neck pain typically been the last week? 0= no bother, 10= worst bother imaginable” (hereafter ‘pain intensity’). A third SMS was sent to all patients “How many days the last week has your neck limited your daily activities? Please answer with a number between 0 and 7.” If the patient failed to answer the weekly SMS, they received a reminder after 2 days. The patient received a verbal reminder by telephone should they miss answering two consecutive weekly SMS.

#### Patient reported baseline variables

Baseline questionnaire (Additional file 2) included age, gender, education level (Primary school, High school, University/higher education ≤4 years, University/higher education > 4 years), as well as paid employment (yes/no), on sick-leave (yes/no), and daily dysfunction (“In your usual daily activities, how much trouble do you have from your neck complaints?” score ranging from 0 = no trouble to 10 = maximal trouble) [20]. Pain intensity was reported as “pain right now” on an 11-point numerical rating scale (NRS, 0–10, where 0 = no pain and 10 = as painful as it is possible to be) [21]. Disability was measured by the Neck Disability Index (NDI) (0 = no impairment to 50 = complete impairment) [22]. The 10 question version of Örebro Musculoskeletal Pain Questionnaire (ÖMPQ) was used for psychosocial screening (0–100), where a higher score is associated with higher risk [23]. General health status was measured on a 0–100 point VAS scale [24], and psychological state and distress was calculated as an average score on the Hopkins Symptom Checklist (HSCL-10) (scores from 1 = not bothered at all, to 4 = very much bothered) [25]. We used a cut-off value above 1.85, which has been proposed for the presence of psychological distress in a Norwegian population [26]. Concomitant musculoskeletal pain was reported by the Nordic Pain Questionnaire (NPQ) [27] and used as follows: Headache (yes/no), low back pain (yes/no), and number of pain sites ≥3 out of 10 (yes/no). Additionally, information regarding pain duration of current NP (0–2 weeks, 2–4 weeks, 1–3 months, 3–6 months, 6 months–1 year, > 1 year), first-time consultation with chiropractor (yes/no), acute onset of pain (yes/no), previous episodes (0, 1–2, and ≥ 3) was collected.

### Data handling

We replicated all data handling, descriptive definitions of subgroups, protocol and coding described below in accordance with the procedures in the Danish LBP cohort [9]. First, we calculated the mean pain intensity and mean paindays from the weekly SMS across the 43 weeks for each patient. This formed the basis for defining the clinical course and the subsequent subgrouping. We subsequently calculated the number of weeks and frequency with deviations of ±1 from the mean pain intensity, as well as the duration and frequency of pain-free weeks. To ensure the best possible comparison between the two studies and the possibility to analyze patterns during the more stable period, we excluded data collected in the first 9 weeks as in the Danish study. Hence, the study period was 43 weeks from week 10 to week 52 in the follow-up.

We imputated missing values on the weekly pain intensity measures in three stages as follows: [1] we replaced missing responses in week 10 (the first week included in the present study) by the equivalent values in week 11 if these were not missing, and similarly, missing responses in week 52 were replaced by the values reported in week 51, [2] we replaced one-week and two-week gaps between weeks with the same pain intensity, with that same value [3]; we excluded from the analysis and categorized patients who after steps 1 and 2 had less than 20 complete responses out of 43 as missing .

#### Categorization into patterns and subgroups

Details of the definitions of patterns and subgroups are shown in Table 1. We modified the nomenclature of the Fluctuating pattern from the original study to improve the understanding. This resulted in four main patterns based on temporal pain variation (hereafter ‘pattern’): Ongoing, Persistent fluctuating, Episodic, and Single episode. Ongoing pattern was the only pattern where the actual number of days with pain per week was defined. Further, patients in the Ongoing pattern should have a variation in pain intensity not exceeding ±1 from the mean value each week. In the Persistent fluctuating pattern, patients should have no pain-free periods of four weeks or more, and variation from the mean pain had to exceed ±1. Patients in the Episodic pain pattern should have pain-free periods of minimum four consecutive weeks between periods with pain. The latter definition was based on previously suggested definitions by de Vet et al., where an episode of LBP is defined as a period of pain lasting more than 24 h, preceded and followed by at least four pain free weeks. Patients with a Single episode could have only one episode lasting 1–2 weeks during the study period. In the present study, the single episode was defined as a short flare-up anywhere during the study period (i.e. after week 9). In addition, the Single episode could not be at the beginning or the end of the study period, as the duration of the episode prior to week 10 or after week 52 would be uncertain.

**Table 1:** Definitions of 4 the main patterns and 16 predefined trajectory subgroups used for analysis as presented in Kongsted et al. [9]

Pattern	Subgroup label	Variation Days	Variation Intensity	Intensity level
**ONGOING**	1. Severe	> 4 days with NP each week	Intensity stays within +/− 1 of mean value	Mean intensity ≥6
	2. Moderate	> 4 days with NP each week	Intensity stays within +/− 1 of mean value	Mean intensity ≥4 and < 6
	3. Mild	> 4 days with NP each week	Intensity stays within +/− 1 of mean value	Mean intensity ≥2 and < 4
	4. Minor/recovery	- no pain-free 4-weeks periods *or* - always pain = 0 (recovered)	Intensity stays within +/− 1 of mean value	Mean intensity < 2
**PERSISTENT FLUCTUATING**	5. Severe	No pain-free 4-weeks periods	Difference between mean and min or max value exceeds 1	Mean intensity ≥6
	6. Moderate	No pain-free 4-weeks periods	Difference between mean and min or max value exceeds 1	Mean intensity ≥4 and < 6
	7. Mild	No pain-free 4-weeks periods	Difference between mean and min or max value exceeds 1	Mean intensity ≥2 and < 4
	8. Minor	No pain-free 4-weeks periods	Difference between mean and min or max value exceeds 1	Mean intensity < 2
**EPISODIC**	9. Severe	Pain-free periods of min. 4 weeks in a row between weeks with pain. Four weeks or more without pain in the beginning or end of the course does not indicate a new episode.		Max intensity ≥6
	10. Moderate	Pain-free periods of min. 4 weeks in a row between weeks with pain. Four weeks or more without pain in the beginning or end of the course does not indicate a new episode.		Max intensity ≥4 and < 6
	11. Mild	Pain-free periods of min. 4 weeks in a row between weeks with pain. Four weeks or more without pain in the beginning or end of the course does not indicate a new episode.		Max intensity ≥2 and < 4
	12. Minor	Pain-free periods of min. 4 weeks in a row, but not always pain = 0. Four weeks or more without pain in the beginning or end of the course does not indicate a new episode.		Max intensity < 2
**SINGLE EPISODE**	13. Severe	One single episode or flare-up lasting 1–2 weeks (which are not the first or the last week of measurement)		Max intensity ≥6
	14. Moderate	One single episode or flare-up lasting 1–2 weeks (which are not the first or the last week of measurement)		Max intensity ≥4 and < 6
	15. Mild	One single episode or flare-up lasting 1–2 weeks (which are not the first or the last week of measurement)		Max intensity ≥2 and < 4
	16. Minor	One single episode or flare-up lasting 1–2 weeks (which are not the first or the last week of measurement)		Max intensity < 2

We split each of the four main patterns into 4 subgroups based on mean (Ongoing and Persistent fluctuating patterns) or maximum (Episodic and Single episode patterns) pain intensity across the 43 weeks: Severe (pain intensity ≥6), Moderate (4 ≤ pain intensity < 6), Mild (2 ≤ pain intensity < 4), and Minor (pain intensity < 2). As Ongoing and Persistent fluctuating patterns are characterized by few or no pain-free weeks, we divided them into intensity subgroups based on deviation from mean pain intensity, with Persistent fluctuating displaying larger variation. In contrast, Episodic and Single episode patterns are characterized by pain episode(s) between pain-free periods, where the highest maximum pain of the episode(s) would better describe the severity of the episode(s) reported during the study period. This resulted in 16 different subgroups in total, and we classified the patients into one of these. The “Minor Ongoing/recovered” subgroup also contained patients that scored zero on pain intensity every week. In addition, we repeated the procedure and classified only the patients recruited at first-time consultation into the same subgroups. We did the latter to assess if these patients distributed differently into the subgroups compared to the whole cohort.

To describe how the pain differed between the subgroups we also calculated mean pain intensity and mean paindays across only the weeks when pain was present, as well as the total number of days and weeks with pain in each subgroup. In addition, we calculated and described the frequency and size of the absolute deviation from the mean pain and the duration and frequency of pain-free periods for the Persistent fluctuating and Episodic patterns.

### Statistical analysis

Descriptive analyses are presented as means with standard deviations (SD) and medians with interquartile range (IQR) or range, for normal and not normal distributed continuous variables, respectively. Categorical data are presented with frequencies and percentages. In addition to the description of the characteristics of the patients in the four main patterns, we made a further distinction between the patients in the eight Persistent fluctuating and Episodic subgroups. We used Chi-square test and Fisher exact test to compare baseline data between patters and subgroups for categorical data. Furthermore, we used Dunn’s post-hoc pairwise comparison test [28] and Bonferroni-Holm correction [29] for comparisons between the four patterns. T-tests were used for the comparison of continuous baseline data between the subgroups with the same intensity level (such as between Severe Persistent fluctuating and Severe Episodic) within the Persistent fluctuating and Episodic patterns. We also used ANOVA with Bonferroni post-hoc pairwise comparison tests for evaluation of continuous baseline data between the four patterns. For all comparisons, *p* < 0.05 was considered statistically significant (two-sided).

#### Robustness to inclusion criteria and pattern definitions

We also made a separate analysis for the patients recruited at first-time consultation to examine if they distributed differently from the cohort, and to assess if our inclusion criteria influenced possible differences in distribution. We did similarly with the inclusion criteria, where we changed the criteria from a minimum of 20 to 10 answers out of 43 SMS. In addition, we repeated the t-test analyses comparing the characteristics of Persistent fluctuating and Episodic subgroups after reducing the Episodic definition of pain-free duration between NP episodes from 4-weeks to 2-weeks, as the subgroups differed only on duration of pain-free periods. We considered the patterns to be robust if the distribution did not change appreciably with inclusion of only the first-time consultation patients.

All analyses were carried out using STATA 16 (StataCorp, Texas, USA).

## Results

**Fig. 1:**
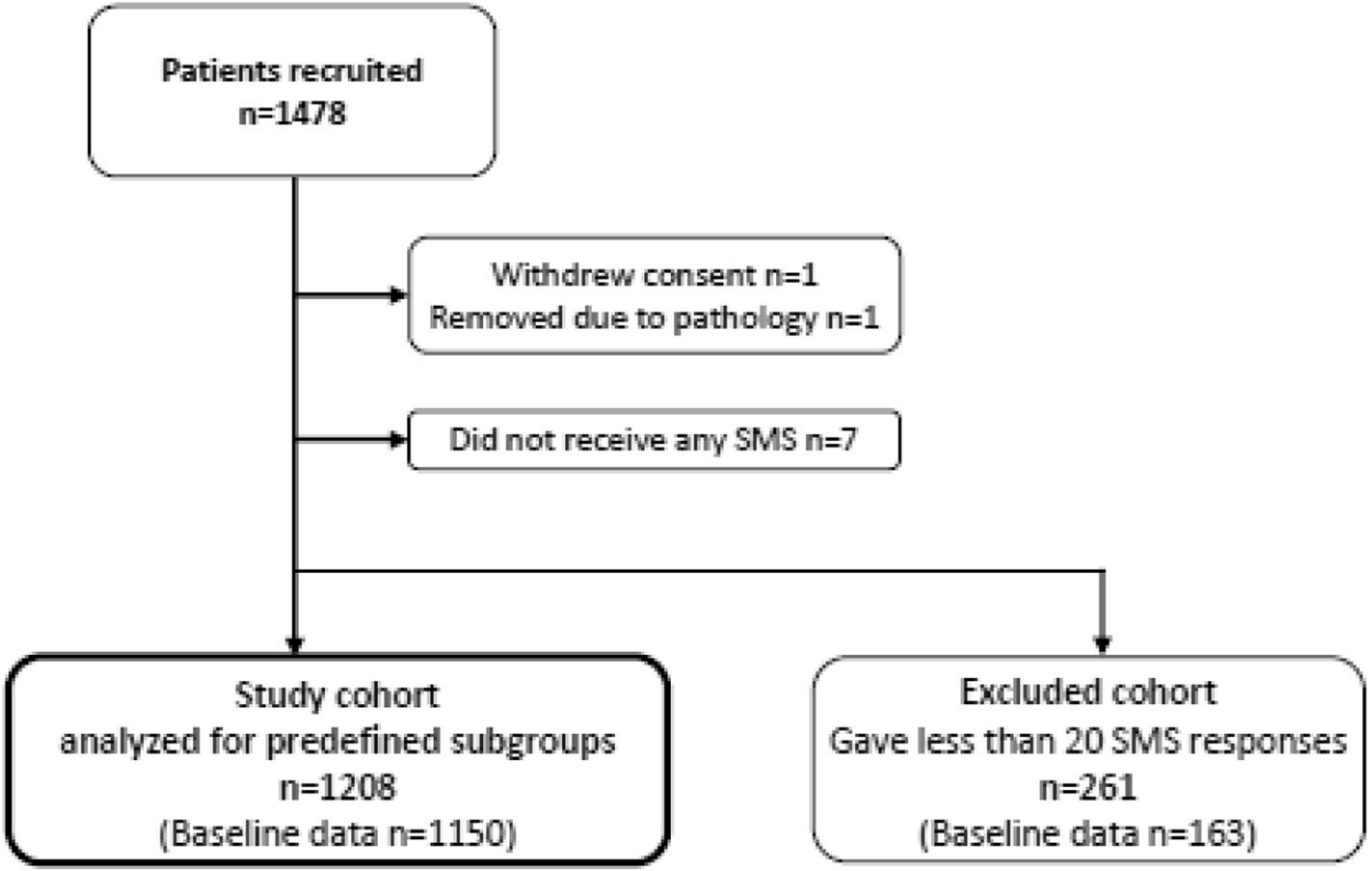
Flowchart recruitment and analysis

A total of 1478 patients consented to participate. One patient withdrew the consent, one was excluded due to being diagnosed with severe pathology after seven weeks of participation, and seven patients did not receive any SMS for unknown reasons. Two-hundred and sixty-one (18%) patients responded to less than 20 SMS follow-ups, and we excluded them from the analyses (the excluded cohort) (Fig. 1). Thus, 1208 patients were available for subgroup analyses. Baseline questionnaires were available from 1150 of these (the study cohort) and from 163 of the excluded cohort.

The patients in the study cohort had a mean (SD) age of 44 [15] years and 74% were female (Table 2). The majority of the patients had experienced NP periods previously and were in an ongoing treatment course. The most common comorbidities were headache, radiating pain to upper extremity, and LBP.

**Table 2:** Characteristics and clinical findings of patients at baseline

Characteristics	Study cohort ***n*** = 1150		Excluded cohort ***n*** = 163	
	n (%)	Mean (SD)	n (%)	Mean (SD)
Age Mean (SD) [range 18–85]		44 (13)		41 (14)
Females	847 (74)		121 (75)	
Radiating pain	859 (76)		127 (79)	
Headache	810 (72)		126 (78)	
Concomitant low back pain	602 (53)		91 (56)	
Number of previous NP episodes				
0	161 (14)		25 (15)	
1–2	197 (17)		24 (15)	
≥3	791 (69)		113 (70)	
First-time consultation with chiropractor	186 (17)		35 (23)	
Duration of NP				
< 1 month	263 (23)		35 (21)	
1–3 months	161 (14)		30 (19)	
> 3 months	710 (63)		97 (60)	
Baseline intensity of NP (NRS 0–10)		4.1 (2.3)		4.5 (2.1)
Disability - NDI (0–50)		12 (6.7)		16 (6.6)
Psychosocial screening - ÖMPQ (0–100)		39 (16)		44 (15)
Psychological distress - HSCL-10 (1–4)		1.6 (0.5)		1.7 (0.5)
General health (VAS 0–100)		71 (19)		69 (21)

*SD* Standard Deviation, *NP* Neck pain, *NRS* Numeric rating scale, *NDI* Neck Disability Index, *ÖMPQ* Örebro Musculoskeletal Pain Questionnaire, *HSCL-10* Hopkins Symptom Checklist-10

The patients in the excluded cohort were younger and slightly more severely affected in terms of disability (NDI) and scored higher on psychosocial screening (Örebro). They did not differ substantially on other parameters (Table 2).

There was an overall high response rate (81–84%), and 55% (*n* = 663) completed all SMS-answers. Eleven percent (*n* = 135) had no pain-free weeks throughout the study period, and 25% (*n* = 301) had no pain-free period lasting more than one week.

### Distribution of NP patients into the defined patterns and subgroups

All but two patients could be classified into one of the defined patterns based on pain intensity and paindays (Table 3). The most common patterns were Persistent fluctuating (48%), and Episodic (45%). The majority of the remaining patients were in the recovered part of the Ongoing/Recovered pattern (4%; all with NRS = 0 each week).

**Table 3:** Distribution of patients in the defined variation patterns and subgroups

Defined patterns and subgroups	Prevalence *n* = 1206	Number of weeks with pain (0–43)	Number of days with pain per week, in weeks with any pain, (0–7)	Pain intensity in weeks with any pain, (0–10)	Total number of days with pain during 43 weeks, (0–301)	
	n (%)	Median (IQR)	Mean (SD)	Mean (SD)	Mean (SD)	Range
**Ongoing**						
1 Severe	1 (0.1)	22	7.0 (0)	6.0 (0)	161 (0)	–
2 Moderate	1 (0.1)	40	7.0 (0)	5.0 (0)	280 (0)	–
3 Mild	0 (0)	–	–	–	–	–
4 Minor/recovered	49 (4.1)	0 (0–0)	0 (0–0)	0 (0–0)	0 (0–0)	0
Total Ongoing	51 (4.2)	0 (0–0)	7.0 (0)	5.5 (0.7)	4.5 (36)	0–280
**Persistent fluctuating**						
5 Severe	54 (4.5)	43 (40–44)	6.0 (1.0)	7.2 (0.9)	252 (51)	116–301
6 Moderate	185 (15.4)	43 (39–44)	4.5 (1.5)	5.0 (0.6)	182 (68)	50–301
7 Mild	298 (25.0)	40 (34–43)	3.3 (1.4)	3.4 (0.6)	130 (66)	29–301
8 Minor	45 (3.9)	34 (26–40)	2.6 (1.4)	2.0 (0.5)	87 (68)	20–301
Total Persistent fluctuating	582 (48.3)	41 (35–43)	3.9 (1.7)	4.1 (1.4)	148 (75)	20–301
**Episodic**						
9 Severe	276 (22.6)	20 (13–27)	2.9 (1.1)	3.9 (1.0)	59 (40)	5–217
10 Moderate	174 (13.9)	14 (8–22)	2.4 (0.9)	2.7 (0.6)	39 (29)	3–153
11 Mild	88 (7.3)	11 (6–18)	2.0 (1.1)	1.9 (0.5)	29 (33)	2–252
12 Minor	9 (0.8)	3 (3–12)	1.5 (0.5)	1.0 (0.0)	13 (13)	1–42
Total Episodic	547 (45.4)	17 (9–25)	2.6 (1.1)	3.2 (1.2)	47 (36)	1–252
**Single episode**						
13 Severe	5 (0.4)	1 (1–1)	3.4 (1.5)	5.8 (1.1)	4.8 (2.6)	1–8
14 Moderate	11 (0.9)	1 (1–2)	2.5 (1.7)	4.0 (0.5)	4.0 (3.6)	1–14
15 Mild	7 (0.6)	1 (1–1)	2.1 (1.2)	2.4 (0.5)	2.3 (1.1)	1–4
16 Minor	3 (0.3)	1 (1–1)	1.0 (0.0)	1.0 (0.0)	1.0 (0.0)	1–1
Total Single episode	26 (2.2)	1 (1–1)	2.5 (1.5)	3.6 (1.6)	3.7 (2.8)	1–14

*IQR* Interquartile range, *SD* Standard deviation

No patients were distributed into the Mild Ongoing subgroup. All patients in the Minor Ongoing/Recovered subgroup were recovered and as such had no days with pain per week and a mean NRS = 0

**Fig. 2:**
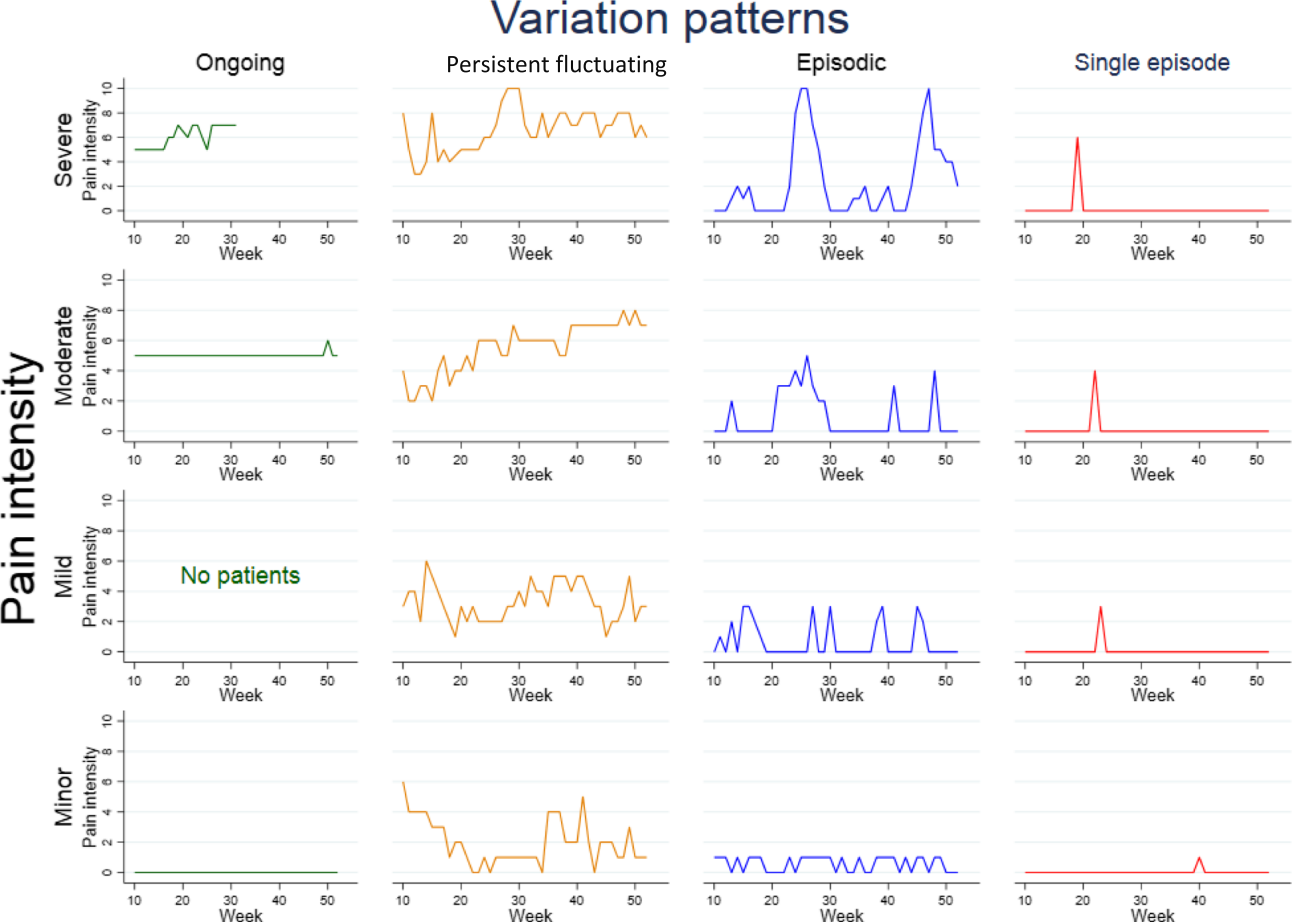
Examples of individual trajectories of the 16 defined subgroups [9], separated into four main patterns

Figure 2 illustrates individual trajectory examples for each of the 16 subgroups. Twenty-five percent of the cohort were classified into the “Mild Persistent fluctuating” subgroup, with a mean (SD) pain intensity of 3.4 (0.6) in weeks with pain and a mean (SD) total number of days with pain of 130 (66) (Table 3). The second most common subgroup was “Severe Episodic” (22%), with a mean (SD) pain intensity of 3.9 (1.0), and a mean (SD) total number of days with pain of 58 [30].

### Exploring characteristics of the patterns

In weeks when pain was present, patients in the Persistent fluctuating patterns reported a higher total number of days with pain, and higher mean pain intensity than patients in the Episodic patterns (Table 3). Patients with pain every week were almost exclusively in the Persistent fluctuating pattern (99.8%). They had smaller variations in pain intensity, but more frequent than those in the Episodic pattern (mean (SD) 1.9 (0.5) vs 2.4 (0.8) points); frequency 18 (range 12-25) vs 13 (range 6-22), respectively).

The Persistent fluctuating and Episodic patterns included significantly more female patients (76 and 74% respectively, *p* ≤ 0.001), patients with pain duration above one month, and less first-time consultation patients. Patients in the Persistent fluctuating pattern scored significantly lower on general health (VAS 66/100), and higher on all other sociodemographic (apart from age and sick leave) and clinical factors than the other three patterns (*p* ≤ 0.006) (Table 4).

**Table 4:** Baseline characteristics and clinical findings of patients in the four variation patterns, and eight subgroups of the Persistent fluctuating and Episodic variation patterns

Main patterns and subgroups NP variables	Ongoing/ Recovered *n* = 45 (3.9%)	Persistent fluctuating *n* = 569 (49.5%)	Episodic *n* = 513 (44.6%)	Single episode *n* = 23 (2.0%)
Age, mean (SD)	47 (16)	45 (13)	44 (12)	41 (13)
Female, n (%)	22 (49)	**424 (76)**^a^	**386 (74)**^a^	14 (61)
Currently on sick leave, n (%)	1 (2.2)	34 (6.1)	26 (5.0)	0 (0)
First episode, n (%)	19 (42)	**53 (10)**^a^	**79 (15)**^a^	9 (39)
Duration > 1 month, n (%)	18 (42)	**484 (88)**^a^	**356 (69)**^a^	11 (50)
> 3 previous episodes, n (%)	14 (31)	**449 (81)**^b^	321 (62)	7 (30)
Concomitant LBP, n (%)	13 (30)	**342 (62)**^b^	240 (47)	6 (27)
Headache, n (%)	18 (42)	**447 (81)**^b^	333 (65)	11 (50)
Pain intensity - NRS (0–10), median (IQR)	3 (1–5)	**5 (3–6)**^b^	3 (2–5)	2.5 (1–4)
Psychosocial screening – ÖMPQ Short form (0–100), mean (SD)	26 (19)	**43 (17)**^b^	33 (16)	24 (16)
General health (VAS 0–100), mean (SD)	80 (13)	**66 (20)**^b^	75 (18)	80 (14)
Psychological distress - HSCL-10, mean (SD)	1.4 (0.4)	**1.7 (0.5)**^b^	1.5 (0.5)	1.3 (0.3)
Severe		**2.0 (0.6)**^c^	1.6 (0.5)	
Moderate		**1.8 (0.5)**^c^	1.5 (0.4)	
Mild		**1.7 (0.5)**^c^	1.5 (0.5)	
Minor		1.5 (0.5)	1.4 (0.2)	
Disability – NDI, mean (SD)	5.2 (3.4)	**13.9 (6.6)**^b^	9.4 (5.9)	7.5 (4.8)
Severe		**22.5 (7.9)**^c^	11.2 (5.9)	
Moderate		**15.5 (5.9)**^c^	8.8 (6.1)	
Mild		**12.1 (5.2)**^c^	7.4 (5.0)	
Minor		**10.1 (5.7)**^c^	5.3 (2.9)	
Radiation into arm at baseline, n (%)	24 (56)	**449 (81)**^b^	371 (73)	12 (55)
Severe		41 (84)	194 (75)	
Moderate		**147 (84)**^c^	115 (70)	
Mild		228 (79)	58 (72)	
Minor		33 (75)	4 (56)	

NP, Neck pain; SD, Standard deviation; LBP, Low back pain; EQ-5D; ÖMPQ, Örebro Musculoskeletal Pain Questionnaire Short-form; IQR, Interquartile range; HSCL-10, Hopkins Symptom Checklist-10; CI, Confidence Interval, 95%; NDI, Neck disability index. ^a,b,c,d^Calculated with Chi^b^ and ANOVA. Results in boldface are statistically significant (*p* < 0.05) differences between: ^a^Persistent fluctuating and Episodic patterns and the Ongoing and Single episode patterns respectively, but not between Persistent fluctuating and Episodic patterns, ^b^Persistent fluctuating pattern and the three other patterns respectively, ^c^ between Persistent fluctuating and Episodic subgroups

Patients in the Persistent fluctuating subgroups scored higher on NDI and HSCL-10 across all intensity levels (*p* < 0.01), apart from the Minor subgroups on HSCL-10 (*p* = 0.29). The proportion of patients above the HSCL-10 cut-off ranged from 55.1% (CI 41.2–69.0) in the Severe Persistent fluctuating subgroup to 23.3% (CI 10.1–35.9) in the Minor Persistent fluctuating subgroup (Table 4).

### Robustness to inclusion criteria and pattern definitions

When limiting the analyses to patients recruited at their first-time consultation with chiropractor for NP (*n* = 186), a slightly lower percentage of patients were classified into the Persistent fluctuating pattern (41.4%) compared to the whole study cohort (48.3%) (Supplementary Table 1, Additional file 3). When we changed the exclusion criteria from responses to minimum 20 to 10 out of 43 SMS, we could have included 42 (3.5%) more patients. There change in distribution was minimal (see Supplementary Table 1, Additional file 3).

When changing the criteria for the Episodic pattern from four to two pain-free weeks between episodes, the number of patients in the Episodic pattern increased from < 1 to 14%, and all were originally classified in a Persistent fluctuating pattern (see Supplementary Table 1, Additional file 3). When calculating the mean pain intensity and number of paindays in the first week following a pain-free period for the whole cohort, only small differences were found when altering the duration of the pain-free period from the recommended 4 weeks to any of one to twenty weeks (see Supplementary Table 2, Additional file 4).

## Discussion

Using a long follow-up period and frequent measurements on a cohort of 1208 NP patients in chiropractic practice, we found that all but two patients could be classified according to the definitions derived from studies of LBP [9]. Most NP patients experienced pain that was either episodic or persistently fluctuating of mild to severe intensity. Steady, persistent pain was almost non-existing in this study cohort of NP patients. Having pain-free periods during the year of follow-up related to a more benign condition concerning dysfunction and psychological distress compared to patterns with more persistent pain.

### Distribution of patterns and subgroups

Using the same pattern definitions as in the Danish LBP cohort [9], 93% of our cohort were classified into Persistent fluctuating and Episodic patterns, compared to 76% in the Danish LBP cohort (see Supplementary Table 3, Additional file 5). In particular, the proportion of NP patients with a Persistent fluctuating pattern was larger than for LBP patients. In general, patients in both cohorts reported quite low pain intensity throughout the study period. However, severe episodic pain was frequent across both cohorts.

These moderate differences in distributions could have several causes, like differences in the two clinical pain conditions or different study designs. The Danish LBP cohort recruited patients from both chiropractic and GP clinics, while our study included chiropractic patients only. The distribution across subgroups in our cohort with NP more closely mirrored the LBP patients from the Danish GP sample [9], which had similar inclusion criteria concerning previous treatment as in our study. The Danish chiropractic sample, however, excluded patients treated by a chiropractor during the last three months prior to inclusion. When compared with our results, this exclusion seems to have reduced the number of patients with a Persistent fluctuating pattern in the Danish chiropractic sample. Even though small differences in distribution was observed between the two cohorts, we found little to no differences in pain intensity and frequency.

The follow-up period in our study differs from previous long-term studies on NP [6, 10]. We excluded the first 9 weeks after inclusion to describe the course of NP in an expectably steadier phase and thus avoid the period after recruitment that is characterized by improvement regardless of previous pain duration or treatment [31, 32]. This makes further direct comparisons between studies difficult. The two other studies identified trajectories based on rapid or slow change from baseline followed by a phase of recovery, with almost three quarters of patients in a “Recovery from mild pain” subgroup. However, where we had only 2 (0.2%) patients with a persistent high pain, the studies of Ailliet and coworkers [6] and Pico-Espinosa and coworkers [10] had to 7 and 11%, respectively. Further, they had none or very few patients in patterns characterized by episodes or persistent pain with intensity variations. It is unclear whether the different findings in the NP studies are due to population or methodological choices like treatment history, differences in sample size and frequency of missing data, or to the fact that different analytical methods are used.

### Robustness of the definitions

To explore the robustness of the definitions, we repeated the classification procedure with altered criteria of the Persistent fluctuating and Episodic patterns. We also applied the definitions to only the group of patients recruited at first-time consultation, as well as to a group where the exclusion criteria was altered from 20 to 10 responses to the 43 weekly SMS. Both approaches resulted in only small differences in distribution into the subgroups.

In contrast to what is expected at the start of a new episode or flare up, there was no increase in pain intensity the first week after a pain-free period ranging from 1 to 20 weeks. In addition, as many as 14% of the patients moved from Persistent fluctuating to Episodic pattern when we changed the definition of an episode from 4 to 2-week pain-free period preceding and following an episode. This indicates that there is a need to further explore and discuss the differences between episodic pain and persistent pain with variations in intensity. The finding can be seen as support to a previously published modified Delphi approach, aiming to standardize LBP recurrence terminology, where concerns were raised about whether timeframes used in definitions of duration of pain and pain-free episodes were arbitrary [33]. The Delphi study defined an episode as follows: with pain intensity of > 2 on an 11-point NRS scale, lasting at least 24 h, and occurring at least 2 times over the past year with at least 30 days pain-free period between episodes [33]. The Episodic pattern used in our study allowed for patients to have only one episode during the follow-up, with pain lasting anything between 3 and 35 weeks. This is in contrast to results from other studies on the course of NP and LBP, where a new episode usually is much shorter and commonly lasts from 2 to 18 weeks [6, 10, 34]. In addition, the definitions used in our study distributed patients with mean pain intensity < 2 into a separate subgroup in each of the four patterns. It could therefore be argued that only 3 intensity levels should be used: Severe (pain intensity ≥6), Moderate (pain intensity 4 ≤ NRS < 6) and Mild (2 ≤ pain intensity < 4), and that patients with pain intensity < 2 should be considered as Recovered.

Our results show that, with the use of the LBP definitions, few NP patients qualified for distribution into the Single episode pattern. The usual curve of improvement from onset of an episode until a more stable pain situation is established, typically lasts 1–2 weeks [34]. For the Single episode pattern definition in our study, the pain could only last 1–2 consecutive weeks followed by completely pain-free weeks. Anything longer, and they were defined as being in the Episodic pattern. The definition criteria, combined with the follow-up period used, could possibly contribute to an increased proportion in the Episodic and decreased the Single episode patterns.

Furthermore, we have not been able to find arguments to support the decision of limiting the number of paindays per week to at least 4 in the Ongoing pattern. Two patients in our cohort fell outside the Ongoing criteria for this reason. Both reported pain every week with pain variation of no more than ±1 from the mean, but having few weeks with 2–4 days with pain each week. Therefore, they neither fitted the Ongoing criteria nor the Persistent fluctuating criteria.

When taking into account the results of the robustness analyses of both our study and the Danish LBP study [9], where five of our 16 subgroups contained less than 5 patients each, there seem to be redundancies in the model. The definitions need further refinement, with possibly combining the Minor subgroups as well as the Ongoing and Persistent Fluctuating patterns. There might also be an idea need to further explore the definitions with regard to number and duration of episodes in Episodic and Single Episode patterns.

### Patient characteristics of patterns and subgroups

Patients in the Persistent fluctuating pattern were distinctly different from the other 3 patterns on all factors except age and sick leave. We found less differences between patients in the Episodic compared to Ongoing and Single episode patterns, apart from the first which had more females. Due to very few patients in the Ongoing and Single episode patterns, these differences should be interpreted with some caution.

Fewer patients in the Persistent fluctuating and Episodic patterns were recruited at first-time consultation, but this is to be anticipated, as these patterns are characterized as being more chronic in both persistency and flare-ups. The vast majority of the patients in the Ongoing pattern were completely recovered in the whole study period (49 out of 51 patients).

Pain intensity and health characteristics followed a similar decrease in severity gradient throughout both Persistent fluctuating and Episodic subgroups. Patients in the Persistent fluctuating subgroups were significantly more distressed and negatively affected in terms of pain, disability and psychological distress than those in the Episodic subgroups, with the exception of the Minor subgroups.

Minor subgroups contained few patients and interpretation of results with regards to those subgroups should be interpreted with caution. While many of the differences are small, the differences in NDI are all above the clinical significance of 30% [35, 36]. The differences in prevalence of high HSCL-10 scores, though statistically significant, show only a maximum 20% difference between the patterns and subgroups. Furthermore, only Severe Persistent fluctuating subgroup was considered clinically meaningful above the cut-off of 1.85, indicating psychological distress [37]. Still, NP episodes separated by periods without pain appears to have considerably less negative impact on daily life. Similar findings have previously been reported for LBP patients [11, 38, 39]. It can be discussed if trajectories and subgroups are condition and/or population specific. They might simply be characteristics of the course of musculoskeletal pain in general [12, 13, 15, 40], and serve as an initial step for decision-making in patient management, irrespective of pain-site and diagnosis [30, 41]. Regardless, our results highlight the need to customize treatment to previous and expected course of pain for patients with NP.

Whether there are different underlying pain mechanisms for these types of patterns is uncertain [42, 43]. The patients in the Persistent fluctuating pattern might represent a more inflammatory--mediated pain [44, 45], and likely to need more comprehensive, multidisciplinary care, while advice and short-term advice might suffice for patients in certain Episodic patterns. Although subgrouping is needed and called for [2, 46], it is also questioned with regard to clinical relevance and usefulness [47]. However, our study strengthens the evidence that terminology like ‘constant’ and ‘intermittent’ are somewhat misleading, as they do not differentiate on the nuances regarding the importance of variation of pain intensity, or duration and frequency of painful and pain-free episodes [48, 49]. Its immediate usefulness seem to be as basis for future studies and as a tool in communicating realistic outcomes and explain probable future course and subsequent management to patients.

### Strengths and limitations

To our knowledge, this is the first study that collected data on treatment-seeking NP patients weekly over 43 weeks, providing evidence on clinical NP development over time. The cohort was large and had a wide geographical distribution. Thus, our data expectably represents NP patients treated by Norwegian chiropractors well. The response rate of SMS was high; between 81 and 84% throughout the study period. Norwegian and Danish populations appear to be rather similar, strengthening the similarities between the two cohorts for comparison purposes.

The chiropractors were asked to invite consecutive patients with NP to limit bias, but we were unable to get usable data for patients who were not invited or declined participation. Selection bias can therefore not be ruled out. We excluded 261 (18%) patients who responded to less than 20 of the 43 SMS to ensure clear-cut subgrouping without imputing data. However, the excluded group did not differ significantly from the analyzed sample suggesting that this did not have substantial influence on the results.

### Future indications

Frequent measures over a long period is time consuming, expensive, and impractical for use in clinical practice. The knowledge of self-reported versus data-driven trajectories is emerging for the LBP population [50, 51], and our study has shown that the same approach can be applied to patients with NP. Although final conclusions cannot be formed from two studies on chiropractic and GP patients, we suggest that reducing the number of subgroups seems logical. It is of clinical interest to explore whether subgroups in general can be used to prioritize patients and identify the need for different types of treatments, and whether this is, in fact, similar across spinal disorders in general.

We excluded data from the first nine weeks as they did in the Danish LBP study, to ensure the comparison of results [9]. It seems, however, relevant to explore if the initial weeks are different to what is considered a more stable period, as the pattern of recovery and relapse in the early phase is often a key factor in treatment planning and prognosis. It is also of interest to explore the stability of the patterns and subgroups over time. Do patients shift between patterns, and do factors like the duration of pain or treatment prior to recruitment influence this? In addition, our study highlights the need to further explore the individual variations in terms of the importance of the duration and frequency of pain-free episodes, as well as further investigation of the difference between duration of a Single Episode/Flare-up versus the Episodic pattern.

## Conclusions

Our study was the first to use defined, standardized definitions of subgroups based on LBP patients in a cohort with NP. We found that the definitions were readily applicable to NP patients. Both NP and LBP patients report mostly low pain intensity, and are characterized by persistent pain with variations in intensity or episodic conditions with pain-free periods. Steady, persistent pain was almost non-existing in this cohort. Persistent fluctuating pain indicate a condition that scores higher than the other patterns in terms of reduced function and psychosocial factors irrespective of severity of pain intensity. Thus, neck pain and low back pain appear to share the same trajectories, with similar baseline characteristics being associated with the various trajectories for both conditions. Our results underscore the importance of using both temporal variation and pain intensity when subgrouping patients.

## Supplementary information

**Additional file 1.** English translation weekly SMS questions.

**Additional file 2.** English translation baseline questionnaire.

**Additional file 3: Supplementary Table 1.** Distribution patterns. Distribution of NP patients into pattern and subgroups with 1) original definition criteria, 2) definition of episode duration of two weeks between pain episodes as part of analyses of robustness of pattern and subgroup definitions, 3) patients recruited at first-time consultation for their neck pain only.

**Additional file 4: Supplementary Table 2**. Intensity of symptoms after pain-free period. NP intensity and weekly days with pain in the first week following a pain-free period of 1 to > 20 weeks for analysis of robustness of pattern and subgroup definitions.

**Additional file 5: Supplementary Table 3**. Distribution NP and LBP cohort comparison. Distribution of NP cohort in the defined patterns subgroups and Danish LBP cohort from Kongsted et al. [9].

## Data Availability

The datasets generated and/or analyzed during the current study are not publicly available due to data protection policies, but are available from the corresponding author on reasonable request.

## Abbreviations

SMS: Short Message Service
NP: neck pain
LBP: low back pain
NDI: Neck Disability Index
ÖMPQ: Örebro Musculoskeletal Pain Questionnaire Short-form
VAS: visual analogue scale
NPQ: Nordic Pain Questionnaire
HSCL-10: Hopkins Symptom Checklist Short Form
SD: Standard Deviation
IQR: Interquartile range
CI: Confidence Interval
ANOVA: Analysis of Variance
NRS: Numeric rating scale
