# Supplementary material for: Neck pain patterns and subgrouping based on weekly SMS-derived trajectories
Source: BMC Musculoskelet Disord. 2020 Oct 14;21:678. doi: 10.1186/s12891-020-03660-0 (PMC7559200; doi:10.1186/s12891-020-03660-0)
Supplement: Supplementary file 1 — Additional file 1. English translation weekly SMS questions. [file 12891_2020_3660_MOESM1_ESM.docx]

**SMS questions sent weekly for 52 weeks**

**SMS 1:**

“How many days the last week has your neck been bothersome? Please answer with a number between 0 and 7”

**SMS 2:**

“How intense has your neck pain typically been the last week? 0= no bother, 10= worst bother imaginable”

**SMS 3:**

“How many days the last week has your neck limited your daily activities? Please answer with a number between 0 and 7”
