# Supplementary material for: Neck pain patterns and subgrouping based on weekly SMS-derived trajectories
Source: BMC Musculoskelet Disord. 2020 Oct 14;21:678. doi: 10.1186/s12891-020-03660-0 (PMC7559200; doi:10.1186/s12891-020-03660-0)
Supplement: Supplementary file 2 — Additional file 2. English translation baseline questionnaire. [file 12891_2020_3660_MOESM2_ESM.docx]

**English translation of baseline questionnaire:**

**Date (ddmmyy):____________________________**

1. **Gender:**

- Female
- Male

**2.How old are you?:**___ ___ years

**3. Weight:**__________kg

**4. Height:**_________cm

**5. Are you a smoker?** (check one box)

- Yes
- No, but I did smoke earlier
- No

**6. Marital status:** (check only one box)

- Married/partnership
- Divorced
- Widowed/widower
- Single

**7. Are you caring for children under the age of 18 on a daily basis?** (check only one box)

- No
- Yes: number of children_______

**Education and employment**

**8. What is your highest completed education?**

(check only one box)

- Elementary school 7-10 years
- Vocational high school, vocational school, secondary school
- High school with general studies
- College or university (less than 4 years)
- College or university (4 years or more)

**9. Your current employment status:** (check all that apply)

- Employed
- Full time
- Part time
- Unemployed
- Pupil/student?
- Retired
- Disabled: ___________%
- Sick-listed : __________%
- By general practitioner
- By chiropractor
- By manual therapist
- Work assessment allowance
- Stay-at-home/not in paid work
- Other______________________________________

**10. How will you describe your current employment?** (check all that apply)

- Mostly sedentary work (e.g. desk work, assembly work)
- Work which require a lot of walking (e.g. clerk work, light industrial work, teaching)
- Work which require a lot of walking as well as lifting (e.g. postman, nurse, kindergarten, construction work)
- Heavy body work (e.g. forestry work, heavy agricultural work, heavy construction work)
- Repetitive work with time pressure
- Self-employed/freelance
- Not in paid work

**11. Describe your current work ability compared to when it was at its best in your life:**

Your best work ability ever is set to 10 points. What number would you put on your present work ability? (Put a circle around the number that best describes your present work ability. 0 points means that you are unable to work at all, whereas 10 points mean that your work ability is currently at its best – please put a circle even if you are not in a paid work situation).

**0 1 2 3 4 5 6 7 8 9 10**

Unable to work work ability

is at its best

**12. Do you enjoy your job, how is your job satisfaction?** (Put a ring around the value that suits you best)

**0 1 2 3 4 5 6 7 8 9 10**

Strongly Thrive very well

dissatisfied

**General health and physical activity**

**13. Other health problems/issues:** (check all that apply)

- no serious or cronic diseases
- cardio vascular diseases
- lung diseases
- gastrointestinal problems
- depression
- urinary tract problems
- diabetes
- cancer
- asthma/allergies
- neurological disorders
- rheumatic disorders
- other musculoskeletal disorders

**14. How often do you exercise?** (take an average)

By exercise/training we mean e.g. walking, cycling, skiing, swimming or other forms of training/sports.

- Never
- Less often than once a week
- Once a week
- 2-3 times a week
- More than 3 times a week

**15. Are you using your bicycle on a daily basis, e.g. as exercise, for leisure activities or as transportation?** (check only one box)

- Yes
- No

**Your neck complaint**

**16. How did your neck complaint occur?** (Check only one box)

- Acute
- Gradually
- I do not know

**17. Did the neck complaint occur as a consequence of:** (check all that apply)

- Physical trauma or injury
- Prolonged load
- Stress-related causes
- Without known triggering cause/Do not know
- Other:____________________________________________

**18. How would you rate your pain right now:** (circle one of the numbers below)

**0 1 2 3 4 5 6 7 8 9 10**

No pain Worst imaginable

pain

**19. How many whole days during the last 4 weeks have you been away from your work due to your neck problems?**

This applies to all absences; self-reported absence as well as sick leave from the doctor (circle only one number).

**0 1 2 3 4 5 6 7 8 9 10 11 12 13 14 15 16 17 18 19 20 21 22 23 24 25 26 27 28**

**Kinesiophobia [6]**

**20. How much fear do you have that these complaints would be increased by physical activity?** (circle only one number)

**0 1 2 3 4 5 6 7 8 9 10**

No fear Very much fear

**21. How do you evaluate the risk for your present complaint developing into persistent problems?** (circle only one number)

**0 1 2 3 4 5 6 7 8 9 10**

No risk Very high risk

**22. Have you had neck complaints previously?** (check only one box)

- No, this is the first time
- Yes, 1-3 times previously

**Last time:__________months ago**

- Yes, more than 3 times previously

**Last time:__________months ago**

- Yes, I do suffer from more or less chronic neck complaints

**23. If the answer is yes, for how many years have you suffered from neck complaints?** (check only one box)

- Less than one year
- 1-5 years
- 6-10 years
- More than 10 years
- As long as I can remember
- I do not know

**Pain medication**

**24. Are you taking pain medication in order to reduce your neck complaint?** (check only one box)

- Never
- No
- Yes:

Over-the counter painkillers (check only one box)

- Never
- Less often than every month
- Every month
- Every week
- Daily
- More times daily

Prescription painkillers (check only one box)

- Never
- Less often than every month
- Every month
- Every week
- Daily
- More times daily

**Treatment for my neck complaint**

**25. What is your goal for the actual treatment of your neck complaint?** (check all that apply)

Pain improvement :

- Become pain free
- Pain reduction
- Excercise without pain
- Prevent pain aggrevation

Improvement of function:

- Improved function in activities of daily living
- Return to earlier activities
- Prevent reduced function

Other goals: _______________________________________________________________________________________

- I do not know/I have no treatment goals

**26.**  **Have you seen any other therapists concerning your current neck complaint** (check only one box)

- No
- Yes

**If the answer is yes,** **what effect did the treatment have on your current neck complaint?** (check all that apply)

|  | Better | Unchanged | Worse |
| --- | --- | --- | --- |
| Physiotherapy with active excercises |  |  |  |
| Physiotherapy with passive treatment modalities (massage, hot packs, electrotherapy an so on) |  |  |  |
| Manual therapy |  |  |  |
| General practitioner |  |  |  |
| Chiropractor |  |  |  |
| Specialist |  |  |  |
| Other treatments |  |  |  |

please specify _______________________________________________

**27. Are you, or have you ever been,** **involved in an insurance claim due to your current or previous neck complaints.** (check only one box)

- No
- Yes, for the current neck complaint
- Yes, for previous neck complaint(s)

**Previous course of pain**

**28.** Below are descriptions of how some people describe their neck pain. Please check the description that you think best represents how your neck pain has been **the previous 12 months.**

| a) |  | No neck pain or just a single episode of neck pain |  |
| --- | --- | --- | --- |
| b) |  | Few episodes of neck pain separated by pain free periods |  |
| c) |  | Mild neck pain most of the time |  |
| d) |  | Neck pain of varying intensity but never completely pain free |  |
| e) |  | Severe neck pain most of the time |  |
| f) | **None of the above illustrated** |  |  |
| g) | **Do not know** |  |  |

**Future course of pain**

**29.**  Below are descriptions of how some people describe their neck pain. Please check the description that you think best represents how your neck pain has been **the next 12 months.**

| a) |  | No neck pain or just a single episode of neck pain |  |
| --- | --- | --- | --- |
| b) |  | Few episodes of neck pain separated by pain free periods |  |
| c) |  | Mild neck pain most of the time |  |
| d) |  | Neck pain of varying intensity but never completely pain free |  |
| e) |  | Severe neck pain most of the time |  |
| f) | **None of the above illustrated** |  |  |
| g) | **Do not know** |  |  |

**In addition to the above questions, patients also completed the following standardized questionnaires:**

**Örebro Musculoskeletal Pain Screening Questionnaire [4]**

**Neck Disability Index (NDI) [5]**

**Hopkins symptom Checklist (HSCL-10) [1]**

**Health-related Quality of Life (EuroQol) [2]**

**Nordic Pain Questionnaire (NPQ) [3]**

[1] Derogatis LR, Lipman RS, Rickels K, Uhlenhuth EH, Covi L. The Hopkins Symptom Checklist (HSCL): a self-report symptom inventory. Behav Sci 1974;19(1):1-15.

[2] EuroQol G. EuroQol--a new facility for the measurement of health-related quality of life. Health Policy 1990;16(3):199-208.

[3] Kuorinka I, Jonsson B, Kilbom A, Vinterberg H, Biering-Sorensen F, Andersson G, Jorgensen K. Standardised Nordic questionnaires for the analysis of musculoskeletal symptoms. Appl Ergon 1987;18(3):233-237.

[4] Linton SJ, Nicholas M, MacDonald S. Development of a short form of the Örebro Musculoskeletal Pain Screening Questionnaire. Spine 2011;36(22):1891-1895.

[5] Vernon H, Mior S. The Neck Disability Index: a study of reliability and validity. Journal of manipulative and physiological therapeutics 1991;14(7):409-415.

[6] Verwoerd AJ, Luijsterburg PA, Timman R, Koes BW, Verhagen AP. A single question was as predictive of outcome as the Tampa Scale for Kinesiophobia in people with sciatica: an observational study. J Physiother 2012;58(4):249-254.
