# Supplementary material for: Neck pain patterns and subgrouping based on weekly SMS-derived trajectories
Source: BMC Musculoskelet Disord. 2020 Oct 14;21:678. doi: 10.1186/s12891-020-03660-0 (PMC7559200; doi:10.1186/s12891-020-03660-0)
Supplement: Supplementary file 3 — Additional file 3: Supplementary Table 1. Distribution patterns. Distribution of NP patients into pattern and subgroups with 1) original definition criteria, 2) definition of episode duration of two weeks between pain episodes as part of analyses of robustness of pattern and subgroup definitions, 3) patients recruited at first-time consultation for their neck pain only. [file 12891_2020_3660_MOESM3_ESM.docx]

**Table S1.** Distribution patterns.

Distribution of NP patients into pattern and subgroups with 1) original definition criteria, 2) definition of episode duration of two weeks between pain episodes as part of analyses of robustness of pattern and subgroup definitions, 3) patients recruited at first consultation for their neck pain only, 4) exclusion criteria: responses <10 out of 43 weeks.

| Patient group | Classified (n=1206) | Classified  (n=1206) | Classified patients recruited at first consultation  (n=186) | Exclusion criteria: responses >10 out of 43 weeks |
| --- | --- | --- | --- | --- |
| Definition criteria | Original | Episode duration 2 weeks | Original | Original |
| Defined patterns and subgroups | n(%) | n(%) | n(%) | n(%) |
| 1 Severe Ongoing | 1 (0.1) | 1 (0.1) | 0 (0) | 0 (0) |
| 2 Moderate Ongoing | 1 (0.1) | 1 (0.1) | 0 (0) | 1 (0.1) |
| 3 Minor Ongoing | 0 (0) | 0 (0) | 0 (0) | 0 (0) |
| 4 Minor Ongoing/Recovered | 49 (4.1) | 49 (4.1) | 10 (4.8) | 58 (4.7) |
| **Total Ongoing** | 51 (4.0) | 51 (4.2) | 10 (4.8) | 59 84.7) |
| 5 Severe Fluctuating | 54 (4.5) | 54 (4.5) | 6 (3.2) | 57 (4.6) |
| 6 Moderate Fluctuating | 185 (15.4) | 156 (13.1) | 23 (12.4) | 196 (15.7) |
| 7 Mild Fluctuating | 298 (25.0) | 192 (15.9) | 42 (22.0) | 304 (24.4) |
| 8 Minor Fluctuating | 45 (3.9) | 15 (1.2) | 7 (3.8) | 50 (4.0) |
| **Total Fluctuating** | 582 (48.3) | 416 (34.7) | 78 (41.4) | 607 (48.9) |
| 9 Severe Episodic | 276 (22.6) | 403 (33.5) | 45 (24.2) | 275 (22.1) |
| 10 Moderate Episodic | 174 (13.9) | 204 (17.0) | 32 (16.9) | 177 (14.2) |
| 11 Mild Episodic | 88 (7.3) | 93 (7.7) | 13 (6.9) | 91 (7.3) |
| 12 Minor Episodic | 9 (0.8) | 10 (0.8) | 1 (0.6) | 7 (0.6) |
| **Total Episodic** | 547 (45.4) | 711 (58.8) | 91 (48.6) | 550 (44.1) |
| 13 Severe Single episode | 5 (0.4) | 5 (0.4) | 1 (0.5) | 7 (0.6) |
| 14 Moderate Single episode | 11 (0.9) | 11 (0.9) | 4 (2.1) | 9 (0.6) |
| 15 Mild Single episode | 7 (0.6) | 7 (0.6) | 1 (0.5) | 10 (0.8) |
| 16 Minor Single episode | 3 (0.3) | 3 (0.3) | 1 (0.5) | 5 (0.4) |
| **Total Single episode** | 26 (2.2) | 25 (2.2) | 7 (3.6) | 31 (2.5) |
| **Not classified n(% of study cohort, n=1208)** | 2 (0.2) | 2 (0.2) | 2 (0.2) | 3 (0.2) |
