# Supplementary material for: Neck pain patterns and subgrouping based on weekly SMS-derived trajectories
Source: BMC Musculoskelet Disord. 2020 Oct 14;21:678. doi: 10.1186/s12891-020-03660-0 (PMC7559200; doi:10.1186/s12891-020-03660-0)
Supplement: Supplementary file 4 — Additional file 4: Supplementary Table 2. Intensity of symptoms after pain-free period. NP intensity and weekly days with pain in the first week following a pain-free period of 1 to > 20 weeks for analysis of robustness of pattern and subgroup definitions. [file 12891_2020_3660_MOESM4_ESM.docx]

**Table S2.** Intensity of symptoms after pain-free period.

NP intensity and weekly days with pain in the first week following a pain-free period of 1 to >20 weeks for analysis of robustness of pattern and subgroup definitions.

| Duration of pain-free periods, weeks | Mean (SD) pain intensity in the first week after a pain-free period, 0-10 NRS | Mean (SD) number of days with NP in the first week after a pain-free period, 0-7 days |
| --- | --- | --- |
| 1 | 3.3 (1.7) | 2.3 (1.4) |
| 2 | 3.3 (1.8) | 2.2 (1.4) |
| 3 | 3.2 (1.8) | 2.2 (1.4) |
| 4 | 3.1 (1.8) | 2.2 (1.5) |
| 5 | 3.1 (1.8) | 2.1 (1.4) |
| 6 | 3.1 (1.5) | 2.1 (1.2) |
| 7 | 3.1 (1.6) | 2.2 (1.5) |
| 8 | 3.1 (1.5) | 2.2 (1.2) |
| 9 | 2.9 (1.7) | 2.3 (1.5) |
| 10-15 | 3.2 (1.6) | 2.3 (1.4) |
| 15-20 | 3.6 (2.0) | 2.6 (1.5) |
| >20 | 3.7 (1.9) | 2.6 (2.0) |
