# Supplementary material for: Neck pain patterns and subgrouping based on weekly SMS-derived trajectories
Source: BMC Musculoskelet Disord. 2020 Oct 14;21:678. doi: 10.1186/s12891-020-03660-0 (PMC7559200; doi:10.1186/s12891-020-03660-0)
Supplement: Supplementary file 5 — Additional file 5: Supplementary Table 3. Distribution NP and LBP cohort comparison. Distribution of NP cohort in the defined patterns subgroups and Danish LBP cohort from Kongsted et al. [9]. [file 12891_2020_3660_MOESM5_ESM.docx]

**Table S3.** Distribution NP and LBP cohort comparison.

Distribution of NP cohort in the defined patterns subgroups and Danish LBP cohort from Kongsted et al (1).

| Defined patterns and subgroups | Prevalence  NP n=1206  LBP n=1077  n (%) | | Number of days with pain per week, in weeks with any pain | | Pain intensity in weeks with any pain | | Total number of days with pain during 43 weeks (301 days) | |
| --- | --- | --- | --- | --- | --- | --- | --- | --- |
|  |  |  | Mean (SD) | | | | | |
| Cohort | NP | LBP | NP | LBP | NP | LBP | NP | LBP |
| 1 Severe ongoing | 1 (0.1) | 3 (0.1) | 7.0 (0) | 6.9 (1.6) | 6.0 (0) | 8.1 (1.6) | 161 (0) | 250 (88) |
| 2 Moderate ongoing | 1 (0.1) | 1 (0.1) | 7.0 (0) | 7 (0) | 5.0 (0) | 4.2 (0) | 280 (0) | 301 (0) |
| 3 Mild ongoing | 0 (0) | 2 (0.1) | - | 6.1 (3.1) | - | 3.0 (1.1) | - | 200 (76) |
| 4 Minor ongoing/ recovered | 49 (4.1) | 155 (14.4) | 0 (0) | 2.2 (2.1) | 0.0 | 1.3 (0.4) | 0 (0) | 2.0 (22) |
| Total Ongoing pattern | 51 (4.0) | 161 (14.9) | 7.0 (0) | N/A | 5.5 (0.7) | N/A | 4.5 (36) | N/A |
| 5 Severe fluctuating | 54 (4.5) | 43 (4.0) | 6.0 (1.0) | 6.1 (1.3) | 7.2 (0.9) | 7.3 (0.9) | 252 (51) | 239 (61) |
| 6 Moderate fluctuating | 185 (15.4) | 87 (8.1) | 4.5 (1.5) | 5.2 (1.5) | 5.0 (0.6) | 5.1 (0.6) | 182 (68) | 206 (69) |
| 7 Mild fluctuating | 298 (25.0) | 113 (10.5) | 3.3 (1.4) | 4.0 (1.7) | 3.4 (0.6) | 3.2 (0.7) | 130 (66) | 155 (81) |
| 8 Minor fluctuating | 45 (3.9) | 22 (2.0) | 2.6 (1.4) | 3.9 (2.1) | 2.0 (0.5) | 1.8 (0.5) | 87 (68) | 143 (90) |
| Total Fluctuating pattern | 582 (48.3) | 265 (24.6) | 3.9 (1.7) | N/A | 4.1 (1.4) | N/A | 148 (75) | N/A |
| 9 Severe episodic | 276 (22.6) | 270 (25.1) | 2.9 (1.1) | 3.3 (1.3) | 3.9 (1.0) | 4.2 (1.2) | 59 (40) | 51 (44) |
| 10 Moderate episodic | 174 (13.9) | 163 (15.1) | 2.4 (0.9) | 2.6 (1.2) | 2.7 (0.6) | 3.0 (0.7) | 39 (29) | 29 (28) |
| 11 Mild episodic | 88 (7.3) | 111 (10.3) | 2.0 (1.1) | 1.9 (1.1) | 1.9 (0.5) | 1.9 (0.5) | 29 (33) | 19 (26) |
| 12 Minor episodic | 9 (0.8) | 8 (0.7) | 1.5 (0.5) | 1.2 (0.4) | 1.0 (0.0) | 1.0 (0.0) | 13 (13) | 6.0 (7.0) |
| Total Episodic pattern | 547 (45.4) | 552 (51.3) | 2.6 (1.1) | N/A | 3.2 (1.2) | N/A | 47 (36) | N/A |
| 13 Severe single episode | 5 (0.4) | 18 (1.7) | 3.4 (1.5) | 4.3 (1.9) | 5.8 (1.1) | 6.4 (1.0) | 4.8 (2.6) | 6.0 (4.0) |
| 14 Moderate single episode | 11 (0.9) | 23 (2.1) | 2.5 (1.7) | 2.4 (1.2) | 4.0 (0.5) | 4.4 (0.6) | 4.0 (3.6) | 3.0 (2.0) |
| 15 Mild single episode | 7 (0.6) | 49 (5.0) | 2.1 (1.2) | 1.8 (1.0) | 2.4 (0.5) | 2.5 0(.5) | 2.3 (1.1) | 2.0 (2.0) |
| 16 Minor single episode | 3 (0.3) | 9 (0.7) | 1.0 (0.0) | 1.1 (0.2) | 1.0 (0.0) | 1.0 (0.0) | 1.0 (0) | 1.0 (1.0) |
| Total Single episode pattern | 26 (2.2) | 99 (9.2) | 2.5 (1.5) | N/A | 3.6 (1.6) | N/A | 3.7 (2.8) | N/A |

NP, Neck Pain; LBP, Low Back Pain; SD, Standard Deviation; N/A, Not Applicable

References

1. Kongsted A, Hestbaek L, Kent P. How can latent trajectories of back pain be translated into defined subgroups? BMC musculoskeletal disorders. 2017;18(1):285.
